# Fifteen Cases of Typhoid Fever in Cook Co. Hospital

**Published:** 1882-03

**Authors:** H. D. Valin


### ARTICLE VII.

FIFTEEN CASES OF TYPHOID FEVER IN COOK COUNTY HOSPITAL. Read Before the Chicago Medical Society, Feb. 6, 1882. Reported by H. D. VALIN, M.D.

I.—O. T., eighteen years of age, a Swede ; laborer ; was admitted to the hospital August 1, 1881, having been sick five weeks already. The disease began with a chill, which recurred three weeks later. Patient had been in bed eleven days. At first there had been constipation, and later diarrhœa. Petechial rose spots were present on the abdomen. On the day of his admission to the hospital, his temperature was 104°, and his pulse 90. He was placed in the wet sheet, took the fever mixture, consisting of cinchonidia sulphate and aromatic sulphuric acid, in ordinary doses, every three hours, and took one-third grain of iodine three times a day from August 16 to 24, when he got well. After August 1 his temperature never rose above 103. He received a milk diet. He was sick eight weeks.

II.—A. Q., æt. twenty ; a Swede ; brick-mason; fell sick July 17, 1881, with a diarrhœa, and had epistaxis the 21st. He was living in bad hygienic surroundings, and he was admitted to the hospital July 25. Temperature 103; pulse 94. His temperature rose to 104 July 30, and to 104.5 August 4, after which it gradually returned to a normal condition, the patient leaving his bed July 20. The high temperature was reduced both times by placing the patient in the wet sheet. The treatment consisted of cinchonidia sulphate and aromatic sulphuric acid. Patient had been sick twelve weeks before entering the hospital, and was not discharged till four weeks later.

III.—J. I. æt. eighteen ; English ; laborer; had been exposed to bad weather, poorly fed, had bad water to drink, and lived in unhealthy surroundings. He was taken sick with chills, fever and diarrhœa, and came to the hospital July 27, the sixth day of his disease. Temperature 103 ; pulse 94. His temperature rose to 104 July 30, and he was placed in the cold wet sheet, cold sheets being renewed every fifteen minutes till the temperature came down to 102 or lower. He received the ordinary dose of cinchonidia sulphate and aromatic sulphuric acid. August 3, his temperature was 104. August 4 his pulse was 62, and his temperature 100.5. He took ten drops of the fluid extract of jaborandi. August 11, his pulse was 58, and his temperature 93.3. He soon afterward got well.

IV.—N. D., æt. twenty-five; Swede; laborer in the lumber yards; was admitted to the hospital August 4, and had been sick already three weeks. He had come to this country nine months ago. Had had epistaxis three times, and diarrhœa. At the time of his admission, his temperature was 103, and it rose to 104 August 6. He was placed in the wet sheet. August 8, it was found necessary to catheterize. Temperature did not rise much above a hundred subsequently, and August 20 he was well. The treatment consisted of the same remedies as in the preceding cases, and he also took one-third of a grain of iodine every three hours.

V.—C. F., æt. twenty-one; Swede; blacksmith; had been sick two weeks when he came to the hospital, August 13. His temperature was 105. His father had died of pneumonia, and he has an œzena. He came to this country seven months ago, and at the time that he was taken sick he slept with nine companions in one room, on Kinzie street. All of them were sick, and four of the number came with him to the hospital, sick with typhoid. August 14 he was kept in the wet sheet three hours, and again the 15th, 16th and 18th. August 23, he was well.

VI.—C. P., æt. twenty-two; Dane; cabinet-maker; was admitted to the hospital August 8, after he had been sick two weeks. He came to this country two months ago, and he had slept with a companion in a small room. Had diarrhœa from the start. The temperature ranged from 100, 102, to 99, and he was discharged August 24. Had the ordinary treatment.

VII.—S. J., æt. twenty-three; Swede; laborer; was sick a week before his admission to the hospital, August 6. He came to this country two years ago. On admission his temperature was 102.5, and there was some diarrhœa. Besides the fever mixture, he took half an ounce of whisky three times a day. His temperature ranged from 102 to 98, and August 27 he was well.

VIII.—F. G., æt. thirty-one; German; willow-ware maker; was sick two weeks before his admission to the hospital, August 11.

His bowels at that time had not moved for four days. Pulse 104; temperature 103.8. Dry, sibilant râles over both lungs, and expectoration of a light-colored mucus. August 22, he took two drachms of castor oil with ten drops of spirits of turpentine, to move his bowels, and took the sulphate of cinchonidia mixture the rest of the time. He was well in a few days.

IX.—E. A., æt. twenty-five; Swede; worked in the lumber yards; was sick a week and a half, and then came to the hospital, August 13, when his pulse was 68, and his temperature 100. His father had pneumonia three times, and he had it himself four years ago. He came to this country fourteen months ago, and had been in Chicago five months. He was taken sick on Kinzie street, with Case V, and several others. He had a chill, and epistaxis. He was administered two drachms of castor oil with ten drops of spirits of turpentine, and received an enema. August 15, his pulse was 48, and his temperature 99. Besides the ordinary mixture, he took five grains of sulphate of cinchonidia every three hours. August 15 and 20 he had a profuse perspiration, and August 22 he was well.

X.—T. M., female, æt. seventeen; Norwegian; came to Chicago a year ago; was sick two weeks before coming to the hospital, August 1. Sickness had begun with a chill, and she was costive. On admission, her pulse was 106, and her temperature was 104.8. Tongue heavily coated brown. There was some sore throat and cough. Sibilant râles over the lungs. She was placed in the wet sheet, but her temperature remained high till August 8. Two small abscesses formed, August 7, on the dorsum of the sacrum, and an eruption appeared all over the body. Patient was somewhat delirious. There was subacute bronchitis. From August 15 patient improved till the 22d, when she got well. There had been no diarrhœa. She took the fever mixture as the rest of the cases.

XI.—J. K., æt. 61; American; machinist; had typhus fever when sixteen years of age. Went to the army, and had chronic diarrhœa during three years; was wounded five times. Present sickness began with a chill, slight vomiting, severe griping pain and tenesmus. Passed a tapeworm in June last. He was admitted to the hospital August 16, after a day's sickness, and his pulse was 93, and temperature 100.2. He took iodine, one-third of a grain three times a day. The diarrhœa stopped August 18. August 22 he was well. There had been no rise in the temperature.

XII.—J. L., æt. twenty-nine; Swede; worked in lumber yards; had been sick a week and came to the hospital, August 8. He had come to this country a year and a half ago. Last spring he had a fever which lasted three weeks. The present sickness began with constipation, and a greenish vomit. August 12, the temperature was 103, and he was placed in the wet sheet for four hours. He began taking iodine August 16, had a profuse perspiration the 18th, and he improved till the 27th, when he got well.

XIII.—S. J. N., æt nineteen; Swede; brick-mason; came to this country a year ago; lodged with three companions in one room, and fell sick with diarrhœa two weeks before coming to the hospital, where he was admitted August 10. His temperature was 104. He coughed and expectorated thick sputum, streaked with bright blood (his mother had died of pneumonia). He was wrapped in the wet sheet four hours. August 17, he began taking iodine, and also received enemas of spirits of turpentine. August 19, he had seven stools, for which he took ten grains of bismuth, with one-fourth grain of morphine. August 20, iodine was discontinued. The 24th, his temperature was 96, and he expectorated rusty sputa. Took half a grain of quinine t. i. d. The 25th, he had more diarrhœa, and took bismuth and morphine as before. August 30 he was well.

XIV.—J. K., æt. thirty; Bohemian; was admitted August 10. Had crepitant and subcrepitant râles over the entire lungs. Pulse 118; temperature 100. Took half an ounce of whisky and two drops of tr. of digitalis every three hours; two and a half grains of sulphate of cinchonidia four times a day. August 16, his temperature was 103.6; the 17th it was 104, and he was placed in the wet sheet. There was pleuritic friction on the right side. Took one-fourth grain of morphine three times at suitable intervals. August 22, he began to fail. Took

℞ Camphoræ....................gr. ij.
Strychn.......................gr. 1-120.
Tr. digitalis....................gtt. v.
Spts. frumenti..................℥ss. M.

every two hours.

August 24 he was partially collapsed, and died the next day. No autopsy. He had propably been sick three weeks before coming to the hospital, but had no interpreter.

XV.—J. L., æt. thirty-seven; Canadian; had been sick two weeks, and came to the hospital August 24. Had lost the use of his left leg by a white swelling of the knee, when fifteen years of age. Had diarrhœa a part of the previous winter, and in June. Temperature and pulse on admission were nearly normal, and he was discharged well August 28.

REMARKS.

These fifteen cases were the first of a series of forty-five which the reporter surveyed, with about the same results. The fever mixture consisted of about eight drops of aromatic sulphuric acid, two and a half grains of sulphate of cinchonidia, a drachm of syrup, and half an ounce of water. The sulphate of cinchonidia seemed fully as good as quinine, and was much cheaper. The cold wet sheet seemed to be by far the readiest and best means of controlling high fever, and whenever the temperature reached above 102.8 an oil cloth was spread over the bed, the patient was placed on it stripped, and covered with a wet sheet, just dipped in water as it came from the hydrant. The sheet was renewed every fifteen minutes, until the temperature had come down to 100 or lower. This would take from half an hour to four or five hours.

By inquiring of the leading physicians in Chicago, it was found that they all used the mineral acids and quinine, giving large doses of the latter when the fever rose, but the wet sheet seemed not to have been used in private practice. The reporter used aromatic sulphuric acid, five drops, and sulphate of cinchonidia, two and a half grains, every three hours with good results in a dozen cases of typhoid fever, and the same proved a valuable remedy in controlling chronic diarrhœa. The iodine treatment, tried in several cases, gave a good result, except in a few where it was stopped on account of vomiting.

